# Effects of matric vs osmotic potential changes on *Variovorax beijingensis* transcription

**DOI:** 10.1128/msystems.00924-25

**Published:** 2025-10-02

**Authors:** Jiwoo Kim, Bjorn Shockey, Kirsten S. Hofmockel, Xiaodong Gao, Caroline A. Masiello, Jonathan J. Silberg

**Affiliations:** 1 Department of Biosciences, Rice University 3990, Houston, Texas, USA; 2 Systems, Synthetic, and Physical Biology Program, Houston, Texas, USA; 3 Biological Sciences Division, Pacific Northwest National Laboratory 6865, Richland, Washington, USA; 4 Department of Earth, Environmental and Planetary Sciences, Rice University 3990, Houston, Texas, USA; 5 Department of Chemistry, Rice University 3990, Houston, Texas, USA; 6 Department of Bioengineering, Rice University 3990, Houston, Texas, USA; 7 Department of Chemical and Biomolecular Engineering, Rice University 3990, Houston, Texas, USA; China Agricultural University, Beijing, China

**Keywords:** gene expression, matric potential, microbe, osmotic potential, RNA, soil, stress, transcriptome, *Variovorax*, water potential

## Abstract

Soil water potential, which regulates microbe-available water, is controlled by osmotic and matric potential, which both become more negative as soils dry. While both parameters can independently alter water potential, the genetic mechanisms underlying microbial responses to both are unknown, with potentially different mechanisms available for microbes to respond to these hydrological parameters. To explore microbial responses to matric vs osmotic potential shifts independently, we evaluated the growth and transcription of *Variovorax beijingensis* in soils and liquid cultures of varying water potential. We found that this microbe respires in dilute minimal medium (−240 ± 104 kPa), in liquid medium containing sucrose (−1,323 ± 21 kPa), and in matrices that span a similar pressure range (−183 ± 55 and −1,393 ± 200 kPa). We show that the global gene expression patterns vary significantly across these four conditions, even when the matric potential and osmotic pressure are set to similar values. However, 68% of the differentially expressed genes (DEGs) observed when transitioning osmotic pressure in liquid medium from −240 to −1,323 kPa were also observed when transitioning matric potential from −183 to −1,393 kPa. As osmotic and matric potential approached the plant wilting point, both presented DEGs implicated in amino acid, betaine, and energy metabolism, as well as plant-growth promotion. While a large overlap was observed in the *Variovorax* transcriptional response to shifts in both osmotic and matric potential, the responses were not identical, with matric potential shifts leading to 2.55-fold more genes exhibiting differential expression.

**IMPORTANCE:**

It remains hard to establish how changes in soil water properties affect microbial behaviors that regulate soil health, and the energy with which soil water is held is likely a holistic control on at least some of those microbial behaviors. This energy is controlled by parameters associated with soil salinity (osmotic potential) and texture (matric potential), which both alter bioavailable water by contributing to total soil water potential. To investigate how the transcription of a soil microbe changes when the microbe-available water is altered either by changing soil texture or by changing osmolyte concentrations, we varied osmotic and matric potential individually and performed RNA sequencing. We observe differences in the transcriptome across all conditions analyzed. However, a large set of genes presented similar gene expression changes when osmotic and matric potential approached the plant wilting point, suggesting that these transcriptional changes are independent of the mechanism that alters soil water potential.

## INTRODUCTION

Soil microbes live in a dynamic environment where hydrological extremes are intensifying with climate change (1–6). Wet-dry cycles can trigger increased CO_2_ emissions through spikes of respiration that occur following soil rewetting (7–11). In part, this increase in metabolic activity occurs because microbial growth is stimulated upon soil hydration (12). This hydration response is a well known, but incompletely constrained driver of soil CO_2_ release to the atmosphere. Microbial gene expression also varies during the growth phases following hydration (13). Following hydration, soil microbes initially focus on generating energy and repairing DNA and then adjust their gene expression to acquire carbon and energy (13). Soil studies have also revealed that characteristics of the extracellular environment contribute to the spike in respiration following hydration, including relatively stable characteristics like soil texture and environmental parameters that dynamically change during dry-wet cycles (14), such as the concentration of osmolytes (15), carbon and nitrogen metabolite concentrations (16), and the soil matric potential (17), a property driven by capillary forces. Our understanding of drivers of respiration changes following soil wet-dry cycles remains limited because, in the natural environment, multiple abiotic and biotic parameters simultaneously vary.

The amount of bioavailable water in soil is controlled by soil water potential (Ψ) (18, 19), which affects microbial growth and activity following rewetting (20). The total soil water potential (Ψ_total_) is determined by four parameters (Ψ_total_ = Ψ_*o*_ + Ψ_*m*_ + Ψ_*g*_ + Ψ_*p*_), including osmotic potential (Ψ_*o*_), matric potential (Ψ_*m*_), gravity potential (Ψ_*g*_), and the hydrostatic pressure of the water column (Ψ_*p*_). A recent study highlighted technical gaps that limit our understanding of biological responses to changes in soil water potential (18). *In situ* measurements of water potential are limited (18), and they are rarely performed in parallel with multiomics measurements (21). In addition, there are no studies directly comparing the effects of different pressure parameters on microbial transcription, such as osmotic and matric potential, which both become more negative as soils dry. This gap constrains the data available to study the mechanistic responses of soil microbes to dynamic moisture changes in field studies. Osmotic and matric potential covary with dry-wet cycles in soil since hydration decreases the concentration of dissolved solutes and the interaction of water molecules with soil particles. This makes it challenging to disentangle the individual effects of osmotic and matric potential on microbial behaviors.

Microbial responses to osmotic stress have been intensively studied (22). These responses include synthesizing compatible solutes like proline, trehalose, and betaine (23–25), taking up solutes from the environment (26), and secreting polysaccharides (27, 28). The ecohydrological role of soil texture on plant fitness has been studied in the context of rainfall fluctuations (29), and microbial responses to textured environments have also been investigated. With some microbes, cell viability decreases as matric pressure becomes more negative with soil drying (30, 31), with some microbes presenting a greater sensitivity to matric potential shifts (32), as observed with osmotic stress. Furthermore, studies examining microbial behaviors have revealed that both the soil matrix and hydration level influence phenotypes (33). A wide range of studies has examined the effects of soil water potential on community composition (34–37), as well as their transcriptional responses (38–40). However, these studies have not compared whether changes in osmotic and matric potential individually result in the same changes in community composition and gene expression. Some studies have compared microbial responses to osmotic and matric potential shifts. In one study, the concentrations of microbial metabolites changed more due to increases in osmotic stress compared to matric stress (41). In another study, increased matric stress was more detrimental to microbial respiration than a corresponding change in osmotic pressure (42). While these studies suggest that microbes may cope with variations in water potential arising from osmotic and matrix stress in different ways, the effects of these different stressors on global gene expression have not been rigorously compared for individual soil microbes.

To better understand how soil microbes respond to osmotic and matric potential changes of similar magnitudes, we varied these parameters individually and studied their effects on the plant growth-promoting microbe *Variovorax beijingensis* (43). We targeted a strain from a model soil community that was previously found to support chitin degradation (44). Since soil microbe persistence prior to rewetting is central for biological contributions to the Birch effect, we sought to first understand the pressures where this microbe persists as soils dry. By monitoring respiration, we find that *V. beijingensis* persists across a wide range of soil water potentials when altered by either adding osmolytes or changing soil texture, which alters matric potential. Using RNA sequencing (45), we map the transcriptional response arising from a more negative soil water potential induced by making the osmotic and matric potential more negative. While this analysis revealed significant differences in the transcriptional responses as osmotic and matrix potential approached plant wilting point, a large fraction (68%) of the genes covaried in their differential expression.

## MATERIALS AND METHODS

### Cell growth

*V. beijingensis*, which has the strain designation PNNL_MSC-1, was isolated from a field site in Prosser, WA (44). This strain was grown in a variety of media, including Luria-Bertani (LB) medium, M9 minimal medium, and modified M9 medium (mM9). LB (per liter) was prepared by mixing tryptone (10 g), NaCl (5 g), and yeast extract (5 g). M9 contained M9 salts, glucose (0.4%), MgSO_4_ (2 mM), and CaCl_2_ (0.1 mM). M9 salts were prepared as a 5× stock by mixing KH_2_PO_4_ (15 g), Na_2_HPO_4_·7H_2_O (64 g), NaCl (2.5 g), and NH_4_Cl (5 g) per liter of water. The modified M9 (mM9) was designed to make liquid media with a lower water potential while keeping the concentration of glucose and NH_4_Cl the same concentration as M9 medium. The 5× salt stock was prepared identically, except that no NH_4_Cl was included. Instead, it was added at a concentration of 1 g/L in the mM9. To adjust the water potential of mM9 media, two approaches were used. Either the concentration of salt stock added was varied, or sucrose was added. LB medium, 5× M9, and mM9 salt stocks were all autoclaved, while other growth medium reagents were sterile filtered through a 0.2 µm filter (Thermo Scientific and Satorius AG). Chemicals for making growth media were from Sigma-Aldrich and Thermo Scientific.

### Artificial soil preparation

Artificial soils were produced using a previously described approach (31). Briefly, nonreactive quartz materials of three different particle sizes (whole grain fine quartz sand with particle size ~70 µm, ground silt-sized quartz with particle size ~8.71 µm, and clay-sized quartz with particle size ~1.7 µm obtained from US Silica) were used to represent soil particles of sand, silt, and clay, respectively. By varying the percentage of the three quartz particles, we produced artificial soils with desired soil textures. In addition to soil texture, soil mineralogy also affects soil matric potential (46). Therefore, two common clay minerals, kaolinite and montmorillonite (Spectrum Chemical MFG Corp), were also used to represent a more chemically reactive clay fraction of the artificial soils. Three of these soils were quartz based and varied in texture only, including sand (90% sand, 5% silt, and 5% clay), silt loam (20% sand, 60% silt, and 20% clay), and clay (20% sand, 20% silt, and 60% clay); these are named Quartz-1 (Q1), Quartz-2 (Q2), and Quartz-3 (Q3), respectively. Two additional silt loam soils were created, which contain the clay minerals montmorillonite (M2; 20% sand, 60% silt, and 20% montmorillonite) and kaolinite (K2; 20% sand, 60% silt, and 20% kaolinite).

### Water potential analysis

The water potential of growth medium and hydrated soils was measured using a WP4C dew point potentiometer (METER Group) at room temperature. The dew point potentiometer measures the total water potential of a sample and does not differentiate osmotic and matric potential. The WP4C was calibrated using a 0.5 M potassium chloride standard (AQUALAB by Decagon) to a pressure of −2.20 MPa. All measurements were made using three replicates using precision mode. The dry weight of each artificial soil was hydrated to θ = 0.1 g/g with mM9 and allowed to equilibrate overnight prior to measurement.

### Growth in liquid medium

Colonies of *V. beijingensis* were obtained by growing on LB-agar plates for 72 hours at 30°C. Single colonies were used to inoculate M9 medium (3 mL), which was grown at 30°C while shaking at 250 rpm for 72 hours. The resulting precultures were used as inoculants for all subsequent cultures. To obtain growth curves, the preculture was washed with each medium (1 mL) used in subsequent growth experiments three times. Cells were then resuspended in growth medium (100 µL) to an optical density (OD) of 0.05 in 96-well plates (Nuclon Thermo Fisher). Cell growth was monitored continuously for 72 hours by measuring the OD every 5 minutes, while shaking (2.5 mm amplitude at 105 rpm) at 30°C using a plate reader (Tecan Spark). To evaluate liquid growth containing varying concentrations of M9 salts and sucrose, samples were treated identically, except they were resuspended to an OD of 0.01 in a total volume of 500 µL in a 96-well deep well plate. Following incubation at 30°C, while shaking at 250 rpm, cultures (200 µL) were transferred to a transparent 96-well plate (Nuclon Thermo Fisher), and OD was measured using a plate reader. To evaluate liquid growth in vials, the preculture was washed three times with mM9 and diluted to an OD of 0.1 with a total volume of 1 mL in a 2 mL glass vial (Thermo Scientific 6ACV11-1PT). Cultures were immediately crimped (Thermo Scientific 6ACC11ST1T) and then incubated statically at 30°C for 72 hours. Following incubation, the headspace CO_2_ was measured. To quantify the number of CFU, each culture was diluted serially using mM9, and an aliquot of each dilution (5 µL) was spotted on mM9-agar plates. After 72 hours at 30°C, colonies were counted. All experiments were performed using three biological replicates unless stated otherwise.

### Soil growth analysis

One gram of each autoclaved artificial soil was placed in gas chromatography-mass spectrometry (GC-MS) vials (2 mL). These soils were then hydrated to 10% water content by adding 100 µL of a *V. beijingensis* culture that had been generated by washing the preculture with mM9 three times and resuspending it in mM9 to an OD of 1, keeping the cell number constant relative to the liquid growth experiments. Vials were crimped and inoculated for 72 hours at 30°C. Headspace CO_2_ was then measured. Following this analysis, cells were hydrated with mM9 and transferred to 15 mL conical tubes to obtain cells to spread on plates. M2 required a higher volume of liquid medium (3 mL) compared to Q2 (750 µL), as it was difficult to pipette up the slurry due to its smaller particle sizes. For each sample, a proportional amount of the slurry was serially diluted using mM9, an aliquot (5 µL) of each dilution was spotted on mM9-agar plates at different dilutions, and plates were incubated for 72 hours at 30°C. Following incubation, CFUs were manually counted on plates. Three biological replicates were performed for each experiment.

### Headspace gas analysis

To monitor CO_2_ produced by cultures in sealed vials, GC-MS was used for headspace analysis following 72-hour incubations. This method necessitated the use of crimped vials, which limit oxygen available for growth, and thus do not represent aerobic conditions that arise in some soil environments. This instrument consists of an Agilent 7890B GC system, a 5977B MS, and a 7693A liquid autosampler equipped with a 100 µL syringe. Headspace gas (50 µL) was injected into DB-VRX capillary column (20 m, 0.18 mm I.D., and 1 µm film) at 50:1 split ratio, and the oven temperature was held at 45°C for 1 minute. MS analysis was performed using selected ion monitoring mode for CO_2_ (MW = 44 and 45). We used Agilent MassHunter Workstation Quantitative Analysis software to quantify the peak area of the major ions and used the minor ions as qualifiers. All CO_2_ levels are reported as relative peak area measured using three biological replicates.

### RNA sequencing

To generate samples for RNA sequencing, four samples were set up within crimped 2 mL vials, including cells in liquid mM9 (1 mL) lacking sucrose at an OD of 0.2, cells in liquid mM9 (1 mL) containing 425 mM sucrose at an OD of 0.2, cells in Q2 (1 g) hydrated to 10% water content with mM9 (100 µL) using cells at an OD of 2, and cells in M2 (1 gram) hydrated to 10% water content with mM9 (100 µL) using cells at an OD of 2. Thus, under each incubation condition, a similar number of cells were used to start cultures in mM9 medium, which varied in sucrose concentration, or in the soil matrix. Following incubation for 72 hours, the cells were lysed open with a bead beater (Biospec Products). The total RNA was then extracted using ZymoBIOMICS RNA Miniprep Kit (Zymo Research) in accordance with the manufacturer’s instructions. The concentration and the quality of the extracted RNA were measured using Qubit 4 Fluorometer (Thermo Fisher Scientific) and spectrophotometer (DeNovix DS-11 FX+), respectively. The extracted RNA was prepared for Next Generation Sequencing using Zymo-Seq RiboFree Total RNA Library Kit (Zymo Research) following manufacturer’s instructions. The prepared library was sent to Azenta Life Sciences where the library sizes were validated with Agilent TapeStation (~560 bp) and sequenced using NovaSeq 6000 XP S4. Each sample condition was performed in six biological replicates to minimize the number of false-negative differentially expressed genes (DEGs) (47). With one of the six mM9 culture replicates, the RNA concentration could not be detected using the TapeStation.

### Differential gene expression analysis

The assembled reference genome and annotations were previously described. Raw paired-end FASTQ files were processed using a custom Python (v3.9.21) pipeline (https://github.com/SilbergLabRice/SeqSorcerer). The pipeline was modified to include parameters specific to this study. Quality trimming and FastQC analysis were performed using Trim Galore v0.6.10 (https://github.com/FelixKrueger/TrimGalore) with the settings “--fastqc,” “--paired,” “--length,” “20,” “--clip_R2,” “15,” “--three_prime_clip_R1,” and “15.” The reference genome was indexed and aligned with HISAT2 v2.2.1 (48). The BAM files were processed with SAMtools v1.9 (49). Transcript counts were quantified via featureCounts from the Subread package v2.0.1 with the parameters, “-a,” gtf_file, “-o,” output_file, “-T,” “2,” “-s,” “2,” “-Q,” “0,” “-t,” “CDS,” “-g,” “transcript_id,” “--minOverlap,” “1,” “--fracOverlap,” “0,” “--fracOverlapFeature,” “0,” and “-p” (50).

After the raw sequencing reads were processed, genes with low total counts (≤10) were eliminated. The data set pairs that were directly compared, e.g., liquid medium having high vs low osmotic pressure, were normalized and evaluated for DEGs using DESeq2 v1.44.0 in R v4.4.1 with Benjamini-Hochberg (BH) adjusted *P*-value capped at 0.05 (51). The results from this analysis are provided as Data set S1. To conduct principal component analysis (PCA), the RNA-seq data were normalized using variance stabilizing transformation (“vst”) and “plotPCA” functions from DESeq2 package. The PCA plot was generated via ggplot2 v3.5.1 (52), and the shaded area represents clustering of biological replicates with 95% confidence using a multivariate *t*-distribution. The pairwise Euclidean distance was calculated with the first two principal components of each sample. Permutational multivariate analysis of variance (PERMANOVA; 999 permutations) test was performed with “adonis2” function using Euclidean distance method in vegan package v.2.7-1 (53).

### DEG pathway analysis

The Kyoto Encyclopedia of Genes and Genomes (KEGG) Orthology (ko) numbers assigned to each gene were extracted from the genome annotation (54). These ko numbers were organized by condition and direction of gene regulation. KEGG module enrichment analysis was performed using the “enrichMKEGG” function of the clusterProfiler package v4.12.6 (55). With the analysis, we referenced the “ko” organism database and used a BH-adjusted *P*-value cutoff of 0.05 and *q*-value of 0.2. All RNA-seq data were visualized using ggplot2 (52). The results from this analysis are provided as Data set S2.

### Data analysis

Average and SD of water potential, OD, and headspace CO_2_ were calculated in R. The *P*-value was generated from two-tailed, unpaired *t*-test in R. The data were visualized using ggplot2 (52). The data sets used in the parametric test were found to be normal using the Shapiro-Wilk test (*P*-value > 0.05).

## RESULTS

### Modulating water potential with osmolytes

**Fig 1:**
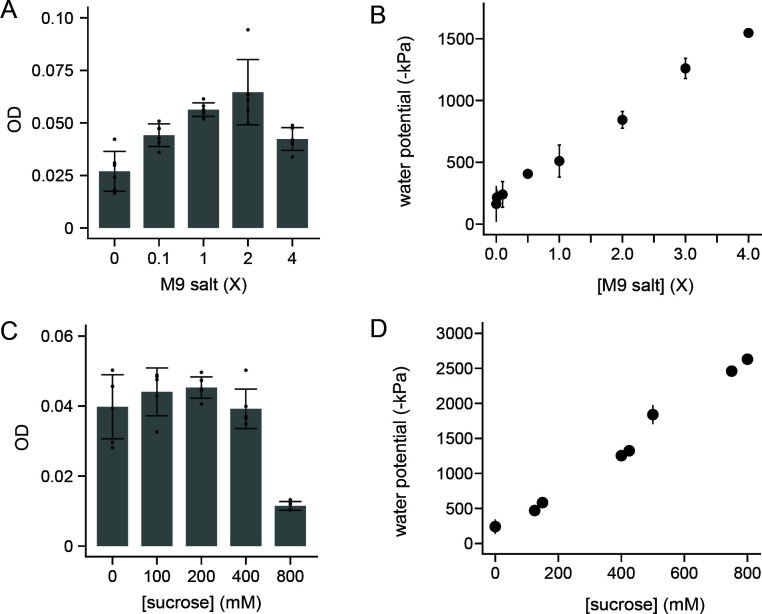
*V. beijingensis* grows across a wide range of osmotic pressures. (**A**) OD of *V. beijingensis* incubated aerobically for 72 hours at 30°C in M9 medium containing varying concentrations of M9 salts while shaking at 250 rpm. (**B**) Water potential of M9 medium containing different concentrations of M9 salts. (**C**) OD of *V. beijingensis* grown in mM9 containing different concentrations of sucrose. Cells were incubated aerobically for 72 hours at 30°C while shaking at 250 rpm. (**D**) Water potential mM9 containing varying concentrations of sucrose. For growth measurements, the points represent the different biological replicates, the bars represent the average, and error bars represent ±1σ. For water potential measurements, points represent the average of three replicates, while the error bars are ±1σ.

*Variovorax* has been implicated as a drought-resistant genus (48, 49, 56), but it is not clear how the persistence of microbes in this genus varies with osmotic pressure. To establish a strategy to tune osmotic pressure in liquid culture and in soils, we varied the osmotic pressure of M9 minimal medium by diluting 5× M9 minimal salts to different extents. With these experiments, the nitrogen (ammonium chloride) and carbon (glucose) sources were held constant at the levels normally found in M9. *V. beijingensis* grew across all conditions analyzed, presenting OD values that increased with M9 salt concentrations (Fig. 1A). When the pressures of each liquid medium were analyzed using a dew point potentiometer (57), this microbe was found to grow across pressures ranging from −150 to −1,500 kPa (Fig. 1B); −1,500 kPa is defined as the permanent wilting point (PWP), representative of the wilting point of most plants (58). These results show that M9 minimal medium, whose salts have been diluted 10-fold (−240 ± 104 kPa), which we call modified M9 medium (mM9), can be used as a frame of reference for well-hydrated soils to study osmolyte and matrix effects on *V. beijingensis* viability. While prior studies have not documented the osmolyte tolerance of this microbe, this water potential represents a value that is not expected to induce stress in the rhizosphere of plants where *Variovorax* is observed (43). This mM9 liquid medium was used for all subsequent measurements.

To alter the osmotic pressure of mM9 medium while minimizing effects on *V. beijingensis* metabolism, we evaluated the effect of adding sucrose on growth and osmotic pressure. This sugar was chosen because *V. beijingensis* cannot use it as a carbon source (44). When cells were provided with sucrose as the only carbon source in M9 medium, growth was not observed (Fig. S1), while robust growth was observed with cells provided with glucose. We next examined how sucrose affects the growth of *V. beijingensis* when added to mM9, which contains glucose. *V. beijingensis* grew to similar final densities in mM9 containing 0–400 mM sucrose (Fig. 1C), although cells presented diminished growth in mM9 containing 800 mM of sucrose (*t*-test; *P* < 0.005). To understand the pressures arising from each sucrose amendment, the potential was measured in mM9 containing different sucrose concentrations using a dew point potentiometer (Fig. 1D). This analysis revealed that *V. beijingensis* grows across in mM9 having pressures ranging from −240 ± 104 kPa (mM9 lacking sucrose) to −1,253 ± 55 kPa (400 mM sucrose). These results show that *V. beijingensis* can grow in a liquid medium presenting a wide range of pressures, induced by adding an osmolyte that this microbe cannot metabolize.

**Fig 2:**
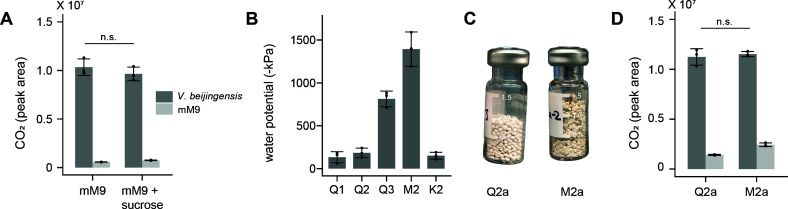
*V. beijingensis* respiration in liquid medium and artificial soils. (**A**) Headspace CO_2_ for *V. beijingensis* grown in crimped vials (dark gray) is compared with cultures lacking cells (light gray). Cells were incubated in mM9 medium containing 425 mM sucrose (−1,323 kPa) or lacking sucrose (−240 kPa) for 72 hours at 30°C without shaking. (**B**) Water potential of artificial soils Q1, Q2, Q3, M2, and K2 hydrated to a water content (θ) of 0.1 g of water per gram of matrix. (**C**) The artificial soils that were hydrated with mM9 containing *V. beijingensis* at a water content of 10% to study the effects of matric potential on respiration. (**D**) Headspace CO_2_ for *V. beijingensis* following 72 hours in each matrix at 30°C. Soils inoculated with cells (dark gray) were compared with those hydrated with mM9 lacking cells (light gray) for each matrix. In all experiments, points represent biological replicates, bars represent the average of all replicates, and error bars represent ±1σ. *P*-values were calculated using two-tailed, unpaired *t*-test (ns, *P* > 0.05).

To investigate if *V. beijingensis* can persist close to PWP (−1,500 kPa), the total CO_2_ respired and the CFU of *V. beijingensis* were measured after incubating cells statically for 72 hours at 30°C in mM9 medium lacking (−240 ± 104 kPa) and containing 425 mM sucrose (−1,323 ± 21 kPa). This high sugar concentration was used to induce osmotic stress, which has been shown to alter global gene expression in other microbes (22). The total CO_2_ accumulation in both conditions was similar (Fig. 2A). Also, plating cells on mM9-agar plates following each incubation yielded similar CFU in the absence (2.6 ± 2.0 × 10^9^) and presence (4.6 ± 1.6 × 10^9^) of sucrose. These findings show that *V. beijingensis* can persist in mM9 medium spanning osmotic pressures that vary by >1,000 kPa.

### Using artificial soils to tune water potential

Artificial soils can be created that mimic soil physical properties, such as soil water energy, by varying structure (31). To screen for artificial soils that can be used to achieve both low and high matric potential when hydrated to the same water content, we characterized the effects of adding mM9 medium on the soil water potential of five different artificial soils (Q1, Q2, Q3, M2, and K2), guided by water potential curves previously reported for these soils (31). When each soil was hydrated with mM9 medium to 100 mg of water per gram of matrix (θ = 10%), we found that Q1, Q2, and K2 yielded pressures ranging from −133 ± 64 kPa (Q1) to −183 ± 55 kPa (Q2; Fig. 2B). These pressures are similar to those observed with liquid mM9 medium in the absence of matrix (−240 ± 104 kPa; *t*-test; *P* = 0.5). M2 yielded the most negative pressure when hydrated to 10% water content with mM9, yielding a pressure (−1,393 ± 200 kPa) that is similar to the pressure of mM9 containing 425 mM sucrose (−1,323 ± 20.8 kPa; *t*-test; *P* = 0.6). An intermediate pressure (−814 ± 91 kPa) was observed with Q3. Thus, when artificial soils are hydrated to 10% water content using mM9, the M2 matrix presents a pressure that is most similar to the pressure observed in mM9 containing 425 mM sucrose, while Q2 exhibits a pressure that is like the pressure of liquid mM9.

The finding that *V. beijingensis* respires and persists in mM9 medium lacking sucrose and containing 425 mM sucrose suggested that this microbe could also persist in the artificial soils presenting a similar range of pressures. To test this idea, Q2 and M2 were hydrated to 10% water content using mM9 containing *V. beijingensis* in crimped vials (Fig. 2C). The number of cells used for inoculation was identical to that of the liquid medium experiments. However, cells were resuspended in smaller volumes of liquid so that they yielded the desired soil water potential following addition to the soils. After 72 hours of static incubation at 30°C, the CO_2_ produced by soils was evaluated, as well as the number of CFU. *V. beijingensis* presented similar respiration in both soils (Fig. 2D), which mirrored that observed with liquid cultures. In addition, cells extracted following each incubation yielded similar CFU for the Q2 (8.9 ± 2.2 × 10^8^) and M2 (5.9 ± 1.3 × 10^8^) soils. These findings identify soil conditions that can create water potentials that mirror the range of potentials generated by adding sucrose to liquid mM9 medium.

### Individual effects of osmotic and matrix stress on transcription

**Fig 3:**
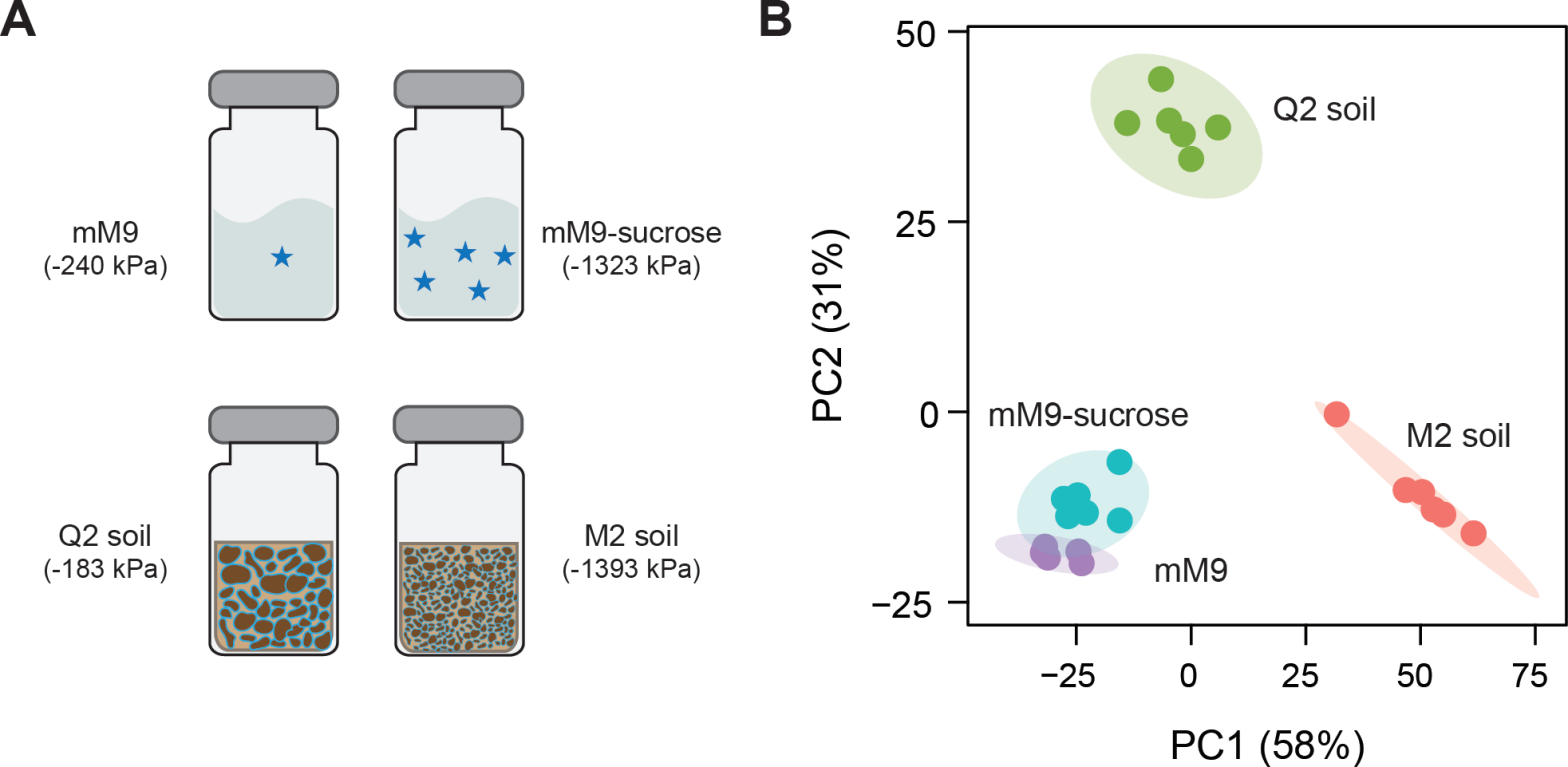
PCA of *V. beijingensis* transcription. (**A**) RNA sequencing was performed on *V. beijingensis* grown in two high-potential conditions, including mM9, having a pressure of −240 ± 104 kPa, and Q2 soil hydrated to 10% water content (−183 ± 55 kPa). In addition, RNA sequencing was performed on *V. beijingensis* grown in two conditions having more negative potentials, including mM9 containing sucrose (−1,323 ± 21 kPa) and M2 soil hydrated to 10% water content with mM9 (−1,393 ± 200 kPa). (**B**) PCA was used to compare the RNA sequencing data across the four different conditions. Each point represents a biological replicate. The shaded clusters for each set of points represent 95% confidence using a multivariate *t*-distribution.

To investigate the effects of osmotic stress on *V. beijingensis* transcription, cells were grown in liquid mM9 medium lacking or containing sucrose (425 mM), total RNA was purified after a 72-hour incubation, and RNA sequencing was performed. In parallel, cells were grown in Q2 and M2 soils hydrated to 10% water content using mM9 medium containing an identical titer of *V. beijingensis*, which yields a similar range of water potentials. Following sequencing, the transcriptomic data for each condition presented a high alignment rate (>80%) to the reference genome, indicating effective rRNA depletion during total RNA purification. To determine if there are significant differences in the transcriptional profiles of cells grown across these conditions, PCA was used to compare the RNA sequencing data. Across both principal components, the separation between the liquid medium treatments presented a smaller separation than between the Q2 and M2 matrix conditions (Fig. 3; Table S1). PERMANOVA using Euclidean dissimilarity revealed significant differences across all conditions and showed that 82% of the total variation is explained by the tested conditions (Table S2). PCA plots for the subsets of significantly upregulated and downregulated genes alone revealed similar differences across the four conditions (Fig. S2). These findings show that all four of the incubation conditions yield distinct patterns of global gene expression, even though some of the conditions have similar water potentials.

**Fig 4:**
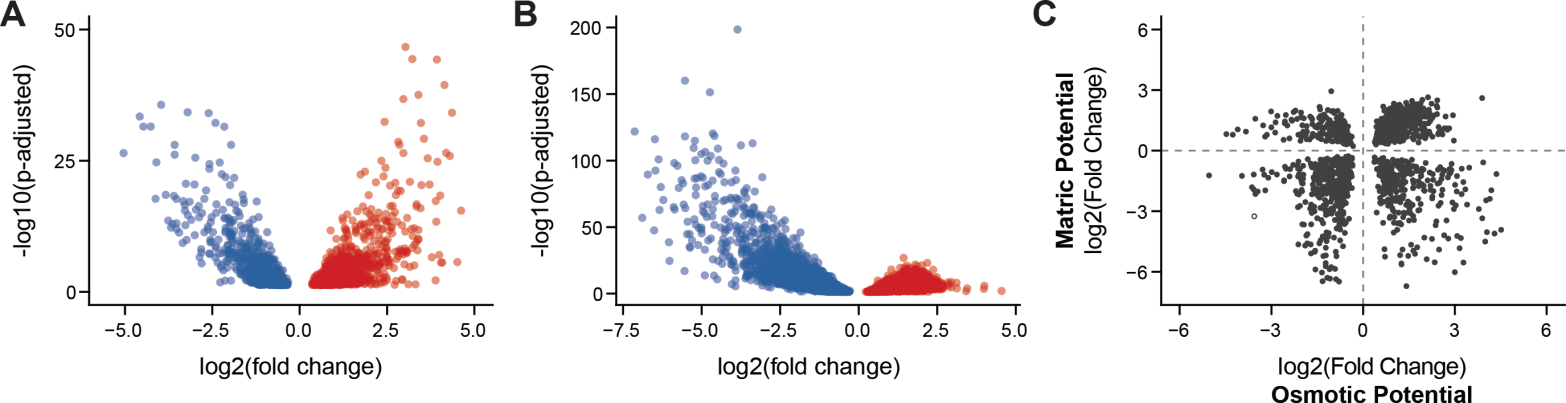
*V. beijingensis* genes that are DEGs when water potential becomes more negative. The changes in gene expression observed when (**A**) comparing cells grown in liquid mM9 medium containing or lacking sucrose and (**B**) comparing cells grown in Q2 and M2 soils hydrated to 10% water content with mM9 medium containing cells. Blue data points represent genes that are downregulated as the soil potential becomes more negative, while red data points represent those that are upregulated as the soil potential becomes more negative. (**C**) A comparison of DEGs arising from similar osmotic and matric potential changes. Among all genes that showed significant changes in gene expression under both osmotic and matric potential treatments (*n* = 1,508 unique genes), approximately two-thirds covaried in the direction of those gene expression changes (*n* = 1,021 genes). Only those genes that presented a significant change in gene expression (Wald test; *P*_adjusted_ <0.05) are shown.

To understand how the gene expression patterns vary with osmotic stress in liquid medium, we evaluated how the gene expression of the 6,440 annotated genes changed in expression in liquid mM9 lacking or containing sucrose (Fig. 4A). The reference genome was previously annotated and filtered for duplicate gene annotations (44). Under osmotic stress conditions (−1,323 kPa), 916 genes were significantly downregulated, while 1,062 genes were significantly upregulated (Wald test; *P*-adjusted < 0.05). To identify pathways that are differentially expressed, an enrichment analysis was performed using the KEGG to evaluate the DEGs presenting significant changes in expression (BH procedure; *P* < 0.05, *q* < 0.2). This analysis revealed that genes implicated in glucose metabolism and oxidative phosphorylation pathways are prominent among those upregulated by osmotic pressure (Fig. S3), including genes involved in glycolysis, the pentose phosphate pathway, gluconeogenesis, and NADH quinone oxidoreductase. Notably, nine genes implicated in betaine metabolism were upregulated under the osmotic stress condition (Table S3), a pathway that has been implicated as an osmotic stress response in other prokaryotes (22, 59, 60). In contrast, pathways implicated in amino acid, nucleotide, and cofactor biosynthesis were downregulated under the more negative osmotic potential condition (Fig. S4). These experiments establish how *V. beijingensis* gene expression changes when cells experience a ~1,000 kPa change in water potential induced by altering the osmotic pressure.

To establish how cells grown in soils having different matric potentials influence gene expression, we investigated how gene expression varies across Q2 and M2 matrices. Under the water potential growth condition of the M2 matrix, which has a pressure that is near permanent wilting point, large numbers of genes were significantly upregulated (*n* = 2,678) and downregulated (*n* = 2,374) compared to cells grown in the Q2 matrix (Fig. 4B). In total, 2.55-fold more genes presented significant changes in gene expression across the two matrices compared with measurements comparing the two liquid growth conditions. When comparing gene expression in M2 to Q2, pathways having large numbers of upregulated genes included those implicated in glucose metabolism, oxidative phosphorylation, and betaine metabolism (Fig. S5). Pathways having the largest numbers of genes downregulated included those implicated in the amino acid biosynthesis, nucleotide metabolism, cofactor synthesis, and the citrate cycle (Fig. S6). These measurements show how gene expression changes when cells experience a ~1000 kPa change in water potential induced by altering the matrix, while holding water content constant.

### Comparing the effects of osmotic and matrix stress

Gene expression measurements revealed that matrix changes induce more DEGs than osmolyte changes (Fig. S7). To better understand how these DEGs relate under the different water potential conditions evaluated*,* the DEGs arising from osmotic and matrix stress were compared (Fig. 4C). In total, 1,508 genes presented a significant change in gene expression when subjected to both matrix and osmotic stress. These DEGs had a large overlap (76%) with the genes that exhibited differential expression in liquid medium. Among these shared DEGs, 1,021 genes (67.7%) covaried in their direction of differential gene expression when cells experienced both osmotic and matrix stress. Thus, although a smaller number of genes present altered gene expression under osmotic stress, a majority of these DEGs present similar responses under matric potential changes of similar magnitude.

**Fig 5:**
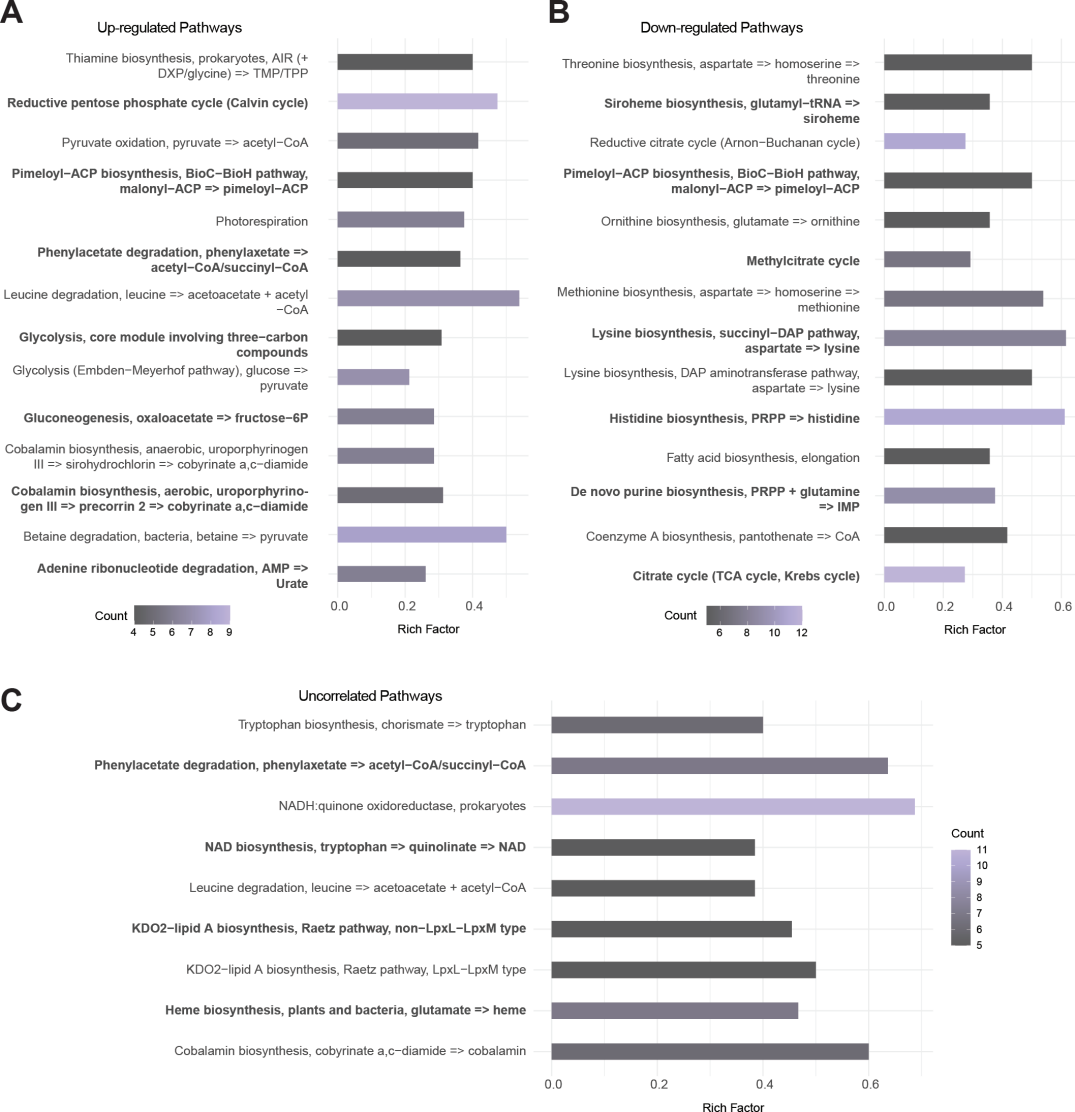
Comparisons of pathways that are differentially expressed under both matrix and osmotic treatments stress. (**A**) DEGs upregulated under both osmotic and matrix treatments. (**B**) DEGs downregulated under both osmotic and matrix treatments. (**C**) DEGs uncorrelated across osmotic and matrix treatments. For each pathway, the fraction of DEGs is noted (Rich Factor), the absolute number of genes upregulated in the pathway is indicated by the symbol size (Count), and the adjusted *P* values are shaded (BH test; *P*-adjusted < 0.05, *q* < 0.2).

To understand the pathways that covaried as osmotic and matric potential became more negative, KEGG enrichment analysis was performed. Metabolic pathways that were upregulated under both mechanisms of water potential changes included pathways implicated in betaine metabolism, amino acid metabolism, and energy production (Fig. 5A). Also, the genes encoding 1-aminocyclopropane-1-carboxylate deaminase and nodulation factor transporter were upregulated in both conditions; these represent plant growth-promoting genes (61). Pathways involved in amino acid biosynthesis were prevalent among the DEGs that were downregulated under both types of stress (Fig. 5B). A much smaller number of pathways had DEGs where the direction of the gene expression change was not correlated (Fig. 5C). Among these pathways, cobalamin synthesis and KDO2-lipid A biosynthesis were upregulated by matrix stress and downregulated by osmotic stress, while leucine/lysine degradation and NADH-quinone oxidoreductase were downregulated by matrix stress and upregulated by osmotic stress. Together, these analyses show that a majority of the DEGs arising from shifts in osmotic and matric potential exhibit similar directions of expression changes. They also identify the metabolic pathways having DEGs that respond similarly to changes in water potential arising from increased osmolyte concentrations and altered soil texture, which makes matric potential more negative.

## DISCUSSION

Inoculation of plants with *Variovorax* relieves drought-induced stress (62, 63), increases biomass and yield (64, 65), and enhances nutrient uptake (66). The results described here show that distinct sets of DEGs arise from altering water potential by changing osmolyte concentration and by changing matrix texture, which both occur as soils dry and *Variovorax* relieves plant stress. Across the two water potential parameters probed, ~2.5-fold more DEGs were observed when changing the matrix compared with altering the osmolyte concentration. Although osmotic pressure changes resulted in a smaller number of DEGs compared with changes in matric potential, 76% of the DEGs observed when adding an osmolyte were also observed when changing the matrix. Furthermore, 67% of the shared DEGs presented the same direction of change. Some of these DEGs have been observed in prior osmotic stress studies (23), which have reported that changes in osmotic pressure alter amino acid, carbohydrate, and energy metabolism as well as the synthesis of compatible solutes. This observation identifies a set of DEGs that arise from changes in water potential, independent of the mechanism by which the water potential is changed. Prior transcriptomic studies have revealed shared effects of different osmolytes on transcription, including salts and organic solutes (23–28). Our study extends this shared response to gene expression changes arising from matrix stress, and it identifies the gene expression changes that are unique to water potential changes arising from growing cells in a textured material.

The increased complexity of the gene expression response across the matrix conditions could arise through multiple mechanisms. First, each matrix may create distinct heterogeneous environments through the interaction with water, as hydration pockets in the different matrices may vary in their partitioning of cells and nutrients (67). In this way, matrices can have different effects on the extent to which microbes can access nutrients, whose diffusion is limited by the connectivity of hydration pockets (33). Second, the matrices may not be perfectly matched in their pH, which can lead to DEG pattern changes (68), even though the medium used for hydration of Q2 and M2 contains the same phosphate buffer. Third, the matrices could affect the bioavailable concentration of chemicals in the mM9 growth medium. Prior studies have shown that soil matrices can differentially sorb apolar molecules, thereby affecting the bioavailable concentration of metabolites (69, 70), cell-cell signals (31), and macromolecules (71, 72). Finally, ions in the growth medium could interact differently with the montmorillonite in M2 and the clay-sized quartz in Q2 because these materials differ in their cation exchange capacities (73). To understand how matrices and growth medium with different properties affect gene expression across a wider range of osmotic and matric potentials, future studies should examine DEGs across a wider range of artificial matrices that are hydrated using medium with defined compositions and osmotic potentials (31).

This study separately considered microbial genetic responses to changes in matric and osmotic potential, two major controls on the ability of microbes to access and use water in the soil environment (18). The separate consideration of these terms is crucial to understanding mechanisms of microbial response to climate stressors (3). In addition, agronomic practices can alter soil salinity and soil moisture separately (74). It is useful to understand how each of these stressors independently affects the behavior of important plant growth-promoting microbes like the *Variovorax* strain studied here. Finally, the genetic responses of organisms like *Variovorax* to shifting matric vs osmotic potential provide new genetic connections to climatically important processes like the Birch effect (7), where the flux of CO_2_ from soil microbial behavior is controlled by the variation in soil moisture, not by the amount.

It is unclear how the trends observed might translate to other soil microbes within the context of their microbiomes, where microbe-microbe and microbe-plant interactions also affect gene expression. Soil microbiome studies have examined how community structure changes under treatments that are expected to alter soil water potential, such as salt stress (34), varying moisture levels (35), and soil amendments (37). Similarly, metatranscriptomics has been performed with microbiomes subjected to changes in soil water content and salinity (38–40). However, these studies have not directly evaluated how community composition and gene expression relate to specific soil water potentials arising from discrete changes in osmotic or matric potential. A recent study used a range of -omics tools to study how a reduced-complexity microbial consortium responds to well-defined changes in moisture and matrix by performing experiments in a glass bead porous medium amended with chitin (33). In this study, which used a model community containing *V. beijingensis* (33), the matrix influenced the microbial phenotypes observed, consistent with our findings with the Q2 and M2 soils having distinct textures. Another study investigating dynamic transcriptional changes, using a model community that includes *V. beijingensis*, observed a temporal shift in gene expression during chitin decomposition (75). Taken together, these studies show that to understand differences in osmotic and matrix stress more deeply, there will be a need to go beyond the static snapshot of gene expression in a single microbe and to examine how microbial communities respond dynamically to similar osmotic and matrix potential changes. Finally, one interesting observation with our measurements was the finding that plant growth-promoting genes are upregulated under both osmotic and matric stress. This finding suggests that as rhizosphere microbes experience pressures approaching the permanent wilting point, they will have beneficial effects on their host independent of the mechanism that leads to that pressure change.

## Data Availability

All RNA-seq data have been submitted to the Gene Expression Omnibus (GEO) under accession GSE299872. The code developed for this study can be accessed at https://github.com/SilbergLabRice/SeqSorcerer.

## SUPPLEMENTAL MATERIAL

Data set S1 msystems.00924-25-s0001.xlsx Differentially expressed genes.

Data set S2 msystems.00924-25-s0002.xlsx KEGG analysis data.

Supplemental Figures and Tables msystems.00924-25-s0003.pdf Figs. S1 to S7; Tables S1 to S4.

ASM does not own the copyrights to Supplemental Material that may be linked to, or accessed through, an article. The authors have granted ASM a non-exclusive, world-wide license to publish the Supplemental Material files. Please contact the corresponding author directly for reuse.
