# Supplementary material for: Effects of matric vs osmotic potential changes on Variovorax beijingensis transcription
Source: mSystems. 2025 Oct 2;10(10):e00924-25. doi: 10.1128/msystems.00924-25 (PMC12542664; doi:10.1128/msystems.00924-25)
Supplement: Supplemental Figures and Tables — Figs. S1 to S7; Tables S1 to S4. [file msystems.00924-25-s0003.pdf]

SUPPORTING INFORMATION

Effects of matric versus osmotic potential changes on *Variovorax beijingensis* transcription

Jiwoo Kim[1], Bjorn Shockey[2], Kirsten S. Hofmockel[3], Caroline A. Masiello[1,4,5], and Jonathan J. Silberg[1,6,7*]

Author affiliations:

1. Department of Biosciences, Rice University, 6100 Main Street, Houston, TX, 77005
2. Systems, Synthetic, and Physical Biology Program, 6100 Main Street, Houston, TX, 77005
3. Biological Sciences Division, Pacific Northwest National Laboratory, Richland, WA 99354
4. Department of Earth, Environmental and Planetary Sciences, Rice University, 6100 Main Street, Houston, TX, 77005
5. Department of Chemistry, Rice University, 6100 Main Street, Houston, TX, 77005
6. Department of Bioengineering, Rice University, 6100 Main Street, Houston, TX, 77005
7. Department of Chemical and Biomolecular Engineering, Rice University, 6100 Main Street, Houston, TX, 77005

*Address correspondence to: Jonathan J. Silberg
Biosciences Department
6100 Main Street
Houston, TX 77005 USA
Tel: 713-348-3849
Email: joff@rice.edu

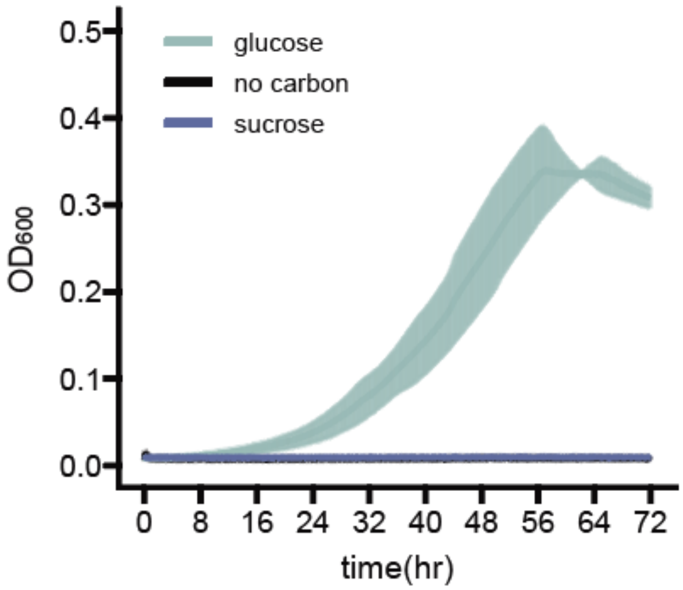

Figure S1. *V. beijingensis* can use glucose but not sucrose as a carbon source. Optical density (OD_{600}) of *V. beijingensis* grown in liquid M9 medium containing either glucose (*green*) and sucrose (*blue*). As a frame of reference cultures grown without a carbon source (*black*) are shown. Cultures were inoculated at a density of 0.05 and grown aerobically at 30°C while shaking at 108 rpm and 2.5 mm amplitude in a plate reader. For each experiment, 3 biological replicates are shown. The lines represent the average, while the shaded region represents ±1 standard deviations.

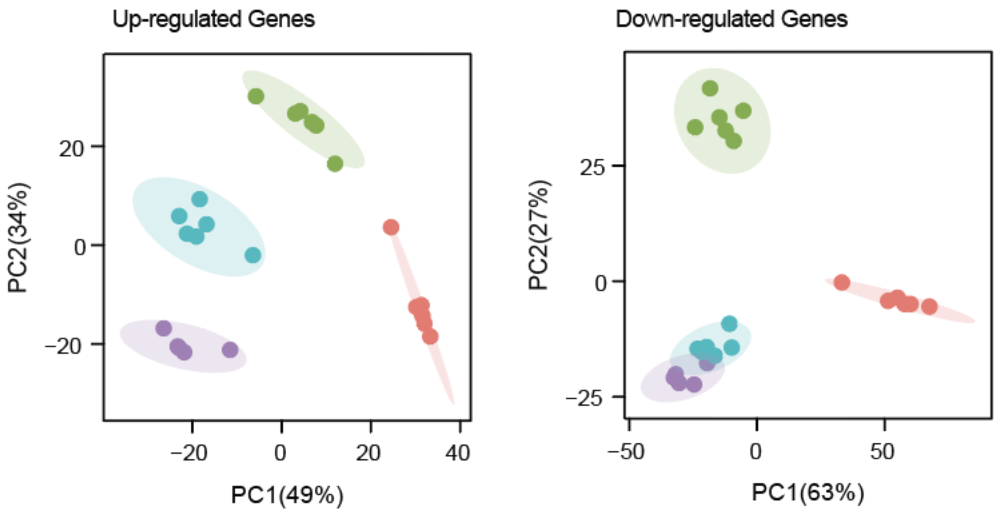

Figure S2. PCA was used to compare the (*left*) upregulated and (*right*) down-regulated genes in the RNA sequencing data across the four different conditions. The four conditions include cells grown in: (i) liquid mM9 medium lacking sucrose, which has a pressure of -240 kPa (purple), liquid mM9 medium containing sucrose with a pressure of -1323 kPa (light blue), Q2 soil hydrated to 10% water content using mM9 which has a matric potential of -183 kPa (green), and M2 soil hydrated to 10% water content with mM9 which has a matric potential of -1393 kPa (red). Each point represents a biological replicate. The shaded clusters for each set of points represent 95% confidence using multivariate t-distribution. All conditions were found to be distinct based on pairwise Euclidean distance (Table S4).

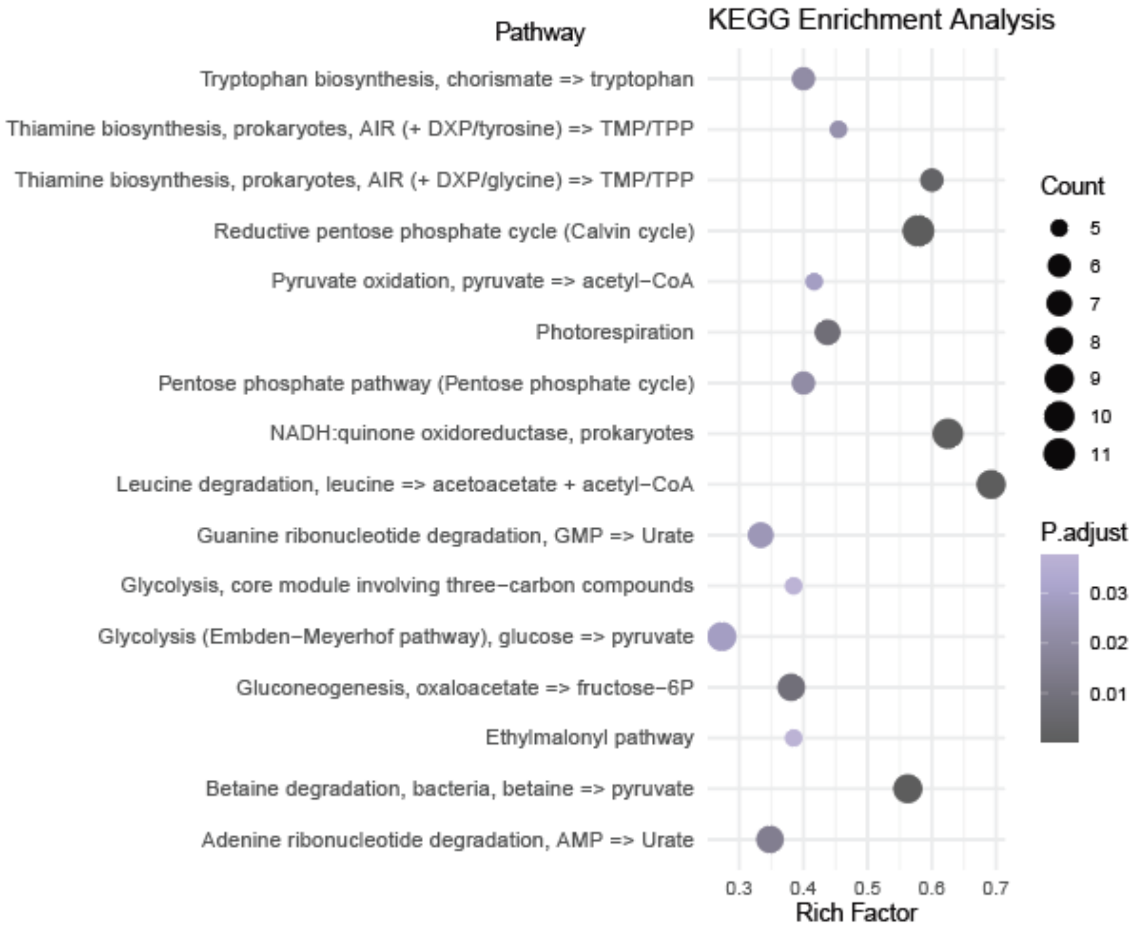

Figure S3. KEGG enrichment analysis of genes upregulated in mM9 medium containing sucrose compared to mM9 lacking sucrose. For each pathway, the fraction of DEGs is noted (Rich Factor), the absolute number of genes upregulated in the pathway is the indicated by the symbol size (count), and the adjusted P values are shaded (BH test; p-adjusted < 0.05, q< 0.2).

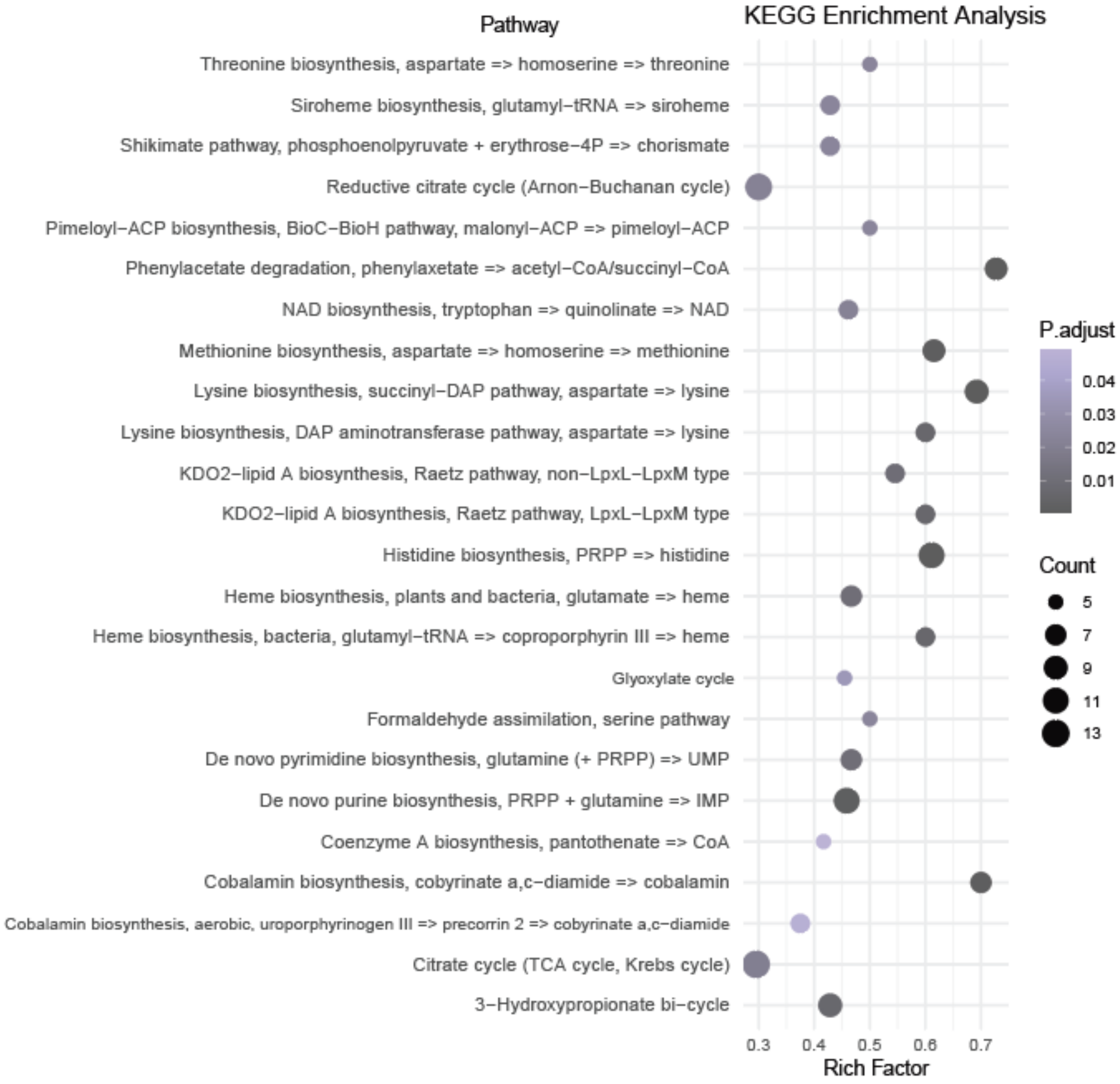

Figure S4. KEGG enrichment analysis of down-regulated genes in mM9 containing sucrose versus mM9 lacking sucrose. For each pathway, the fraction of DEGs is noted (Rich Factor), the absolute number of genes upregulated in the pathway is the indicated by the symbol size (count), and the adjusted P values are shaded (BH test; p-adjusted < 0.05, q< 0.2).

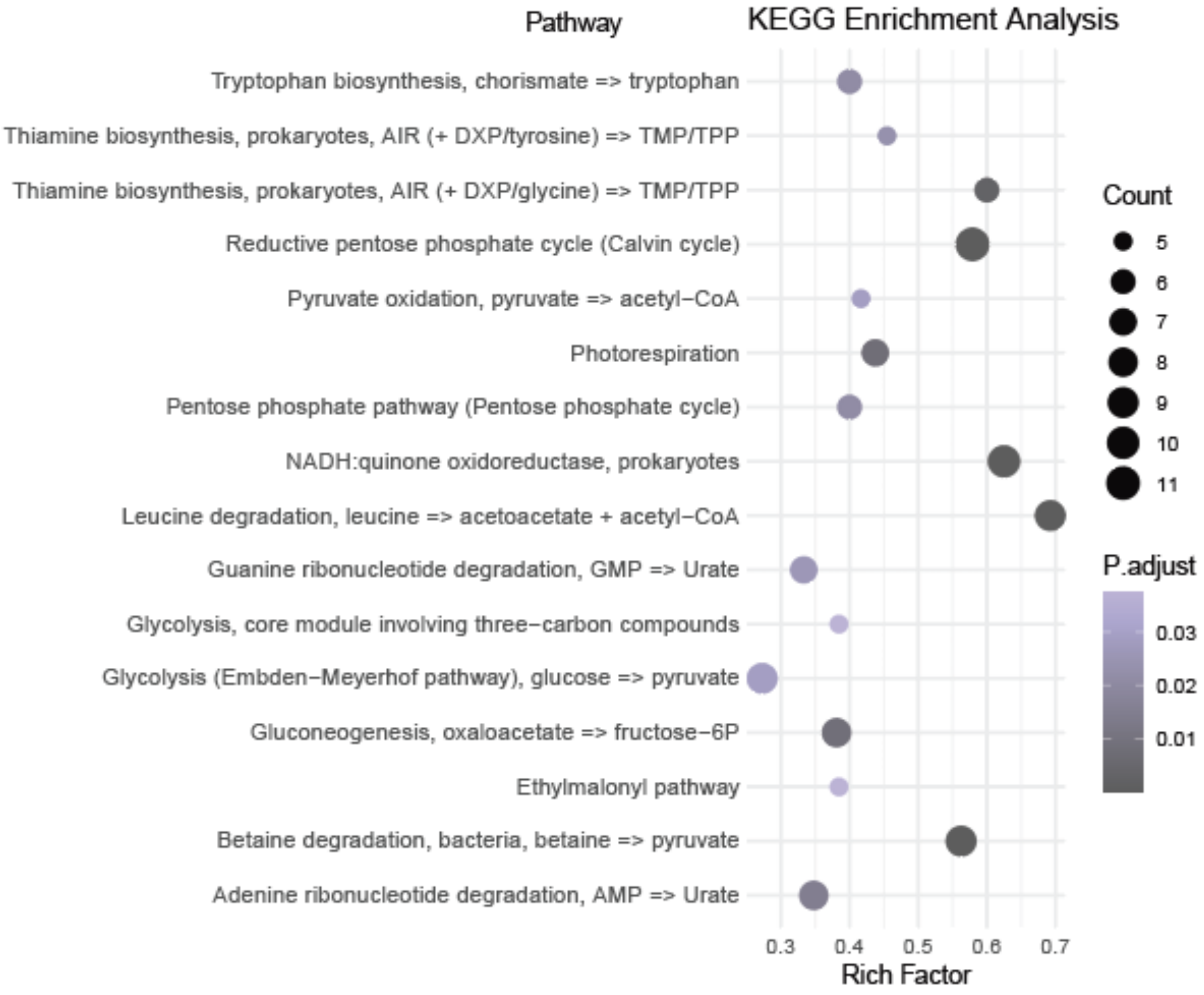

Figure S5. KEGG enrichment analysis of upregulated genes in M2 vs Q2 soil. For each pathway, the fraction of DEGs is noted (Rich Factor), the absolute number of genes upregulated in the pathway is the indicated by the symbol size (count), and the adjusted P values are shaded (BH test; p-adjusted < 0.05, q< 0.2).

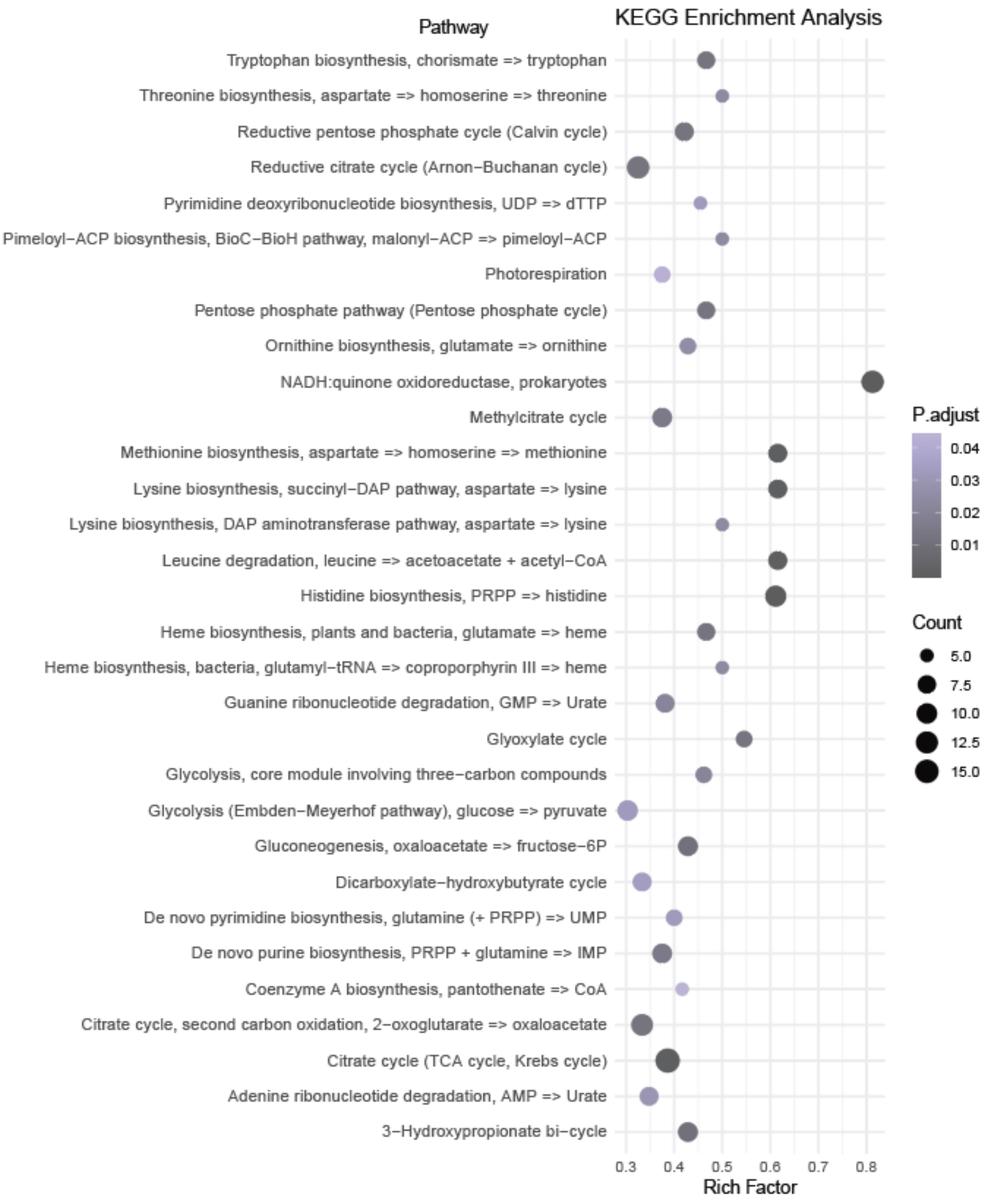

Figure S6. KEGG enrichment analysis of down-regulated genes in M2 vs Q2 soil. For each pathway, the fraction of DEGs is noted (Rich Factor), the absolute number of genes upregulated in the pathway is the indicated by the symbol size (count), and the adjusted P values are shaded (BH test; p-adjusted < 0.05, q< 0.2).

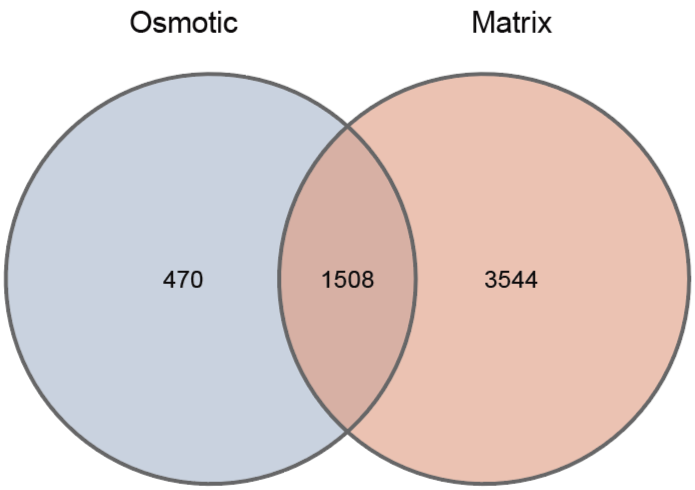

Figure S7. Venn diagram comparing all DEGs observed when altering osmotic and matric potential. Changes in matric potential led to 5052 DEGs, which represent 78% of the 6440 annotated genes in *V. beijingensis*. Changes in osmotic potential led to 1978 DEGs, which represent 30% of the annotated genes.

Table S1. Pairwise Euclidean distance between conditions. Based on the first two principal components calculated from the PCA, pairwise Euclidean distance was examined. The distance is a relative number, and bigger numbers represent greater variability between two groups. RNA sequencing data from measurements in Q2 and M2 soil, respectively. Data from measurements in low (mM9) and high osmolarity (mM9-sucrose) are shown as well.

	**M2 soil**	**Q2 soil**	**mM9-sucrose**	**mM9**
**M2 soil**	0	71.9462	71.9114	78.8553
**Q2 soil**	71.9462	0	52.94916	61.92812
**mM9-sucrose**	71.9114	52.94916	0	9.587551
**mM9**	78.8553	61.92812	9.587551	0

Table S2. Permutational multivariate analysis of variance (PERMANOVA; 999 permutations) of RNA-seq data by condition (mM9-sucrose, mM9, M2 soil, and Q2 soil). The first row “Model” shows statistical test performed by condition, and the second row “Residual” shows test performed by sample. Df represents degrees of freedom, SumSq represents sum of squares, R^2 shows % of total variation explained by the condition, F (F-statistics) is a ratio of inter-group to intra-group variation distance, and Pr(>F) represents *p*-value.

	Df	SumSq	R2	F	Pr(>F)
**Model**	3	97123.49	0.822308	29.30894	0.001
**Residual**	19	20987.3	0.177692	NA	NA
**Total**	22	118110.8	1	NA	NA

Table S3. DEGs in the betaine pathway. The list shows the nine genes implicated in betaine metabolism were upregulated in mM9-sucrose compared to mM9 liquid medium.

Gene	Description	KEGG_ko
G12_RASTassembly.CDS.3181_mRNA	Serine hydroxymethyltransferase	K00600
G12_RASTassembly.CDS.3813_mRNA	Oxidoreductase FAD-binding domain	K21832
G12_RASTassembly.CDS.3820_mRNA	Sarcosine oxidase, gamma subunit family	K00305
G12_RASTassembly.CDS.3821_mRNA	Belongs to the GcvT family	K00302
G12_RASTassembly.CDS.3822_mRNA	Sarcosine oxidase delta subunit	K00304
G12_RASTassembly.CDS.3823_mRNA	FAD dependent oxidoreductase	K00303
G12_RASTassembly.CDS.3824_mRNA	serine dehydratase beta chain	K01752
G12_RASTassembly.CDS.4510_mRNA	PFAM Pyridoxal-5'-phosphate-dependent protein beta subunit	K01754
G12_RASTassembly.CDS.6298_mRNA	4Fe-4S dicluster domain	K21834

Table S4. Pairwise Euclidean distance between conditions. Based on the first two principal components calculated from the PCA of significantly up- and down-regulated genes (Figure S2), pairwise Euclidean distance was examined. The distance is a relative number, and bigger numbers represent greater variability between two groups. RNA sequencing data from measurements in Q2 and M2 soil are shown as well as data from measurements in liquid medium containing mM9 and mM9-sucrose.

**Up-regulated Genes**				
	**M2 soil**	**Q2 soil**	**mM9-sucrose**	**mM9**
**M2 soil**	0	44.71757	50.28334	52.36386
**Q2 soil**	44.71757	0	30.7392	51.98075
**mM9-sucrose**	50.28334	30.7392	0	24.05173
**mM9**	52.36386	51.98075	24.05173	0
**Down-regulated Genes**				
	**M2 soil**	**Q2 soil**	**mM9-sucrose**	**mM9**
**M2 soil**	0	78.57276	71.85817	83.70397
**Q2 soil**	78.57276	0	49.13928	57.38489
**mM9-sucrose**	71.85817	49.13928	0	12.74103
**mM9**	83.70397	57.38489	12.74103	0
